# Real-world gait changes preceding Parkinson’s disease diagnosis in a population-scale UK biobank cohort

**DOI:** 10.1007/s00702-026-03256-3

**Published:** 2026-08-08

**Authors:** Paulo H. S. Pelicioni, Sophie Waller, Lloyd Chan, Arun V. Krishnan, Stephen R. Lord, Matthew A. Brodie

**Affiliations:** 1 School of Health Sciences, University of New South Wales, Randwick, Australia; 2 Neuroscience Research Australia , University of New South Wales , Randwick, Australia; 3 School of Clinical Medicine, University of New South Wales, Randwick, Australia; 4 Department of Neurology , Prince of Wales Hospital , Randwick, Australia; 5 School of Population Health, University of New South Wales, Randwick, Australia; 6 School of Biomedical Engineering, University of New South Wales, Randwick, Australia

**Keywords:** Parkinson, Prodromal, Walking, Wearable, Device, Biomarker

## Abstract

Parkinson’s disease (PD) has a long prodromal phase characterised by non-motor symptoms and subtle motor dysfunction, including changes in gait. While early gait abnormalities have been described in high‑risk populations, less is known about how real‑world gait behaviour changes prior to diagnosis in the general population. To investigate whether digital gait biomarkers derived from wrist‑worn sensors are associated with future Parkinson’s disease diagnosis. The study comprised 73,413 UK Biobank participants who wore a wrist-worn device for seven days. Seventeen digital gait biomarkers were derived using the Watch Walk algorithm. Participants were followed for up to 10 years through linked electronic health records, and associations with time to PD diagnosis were assessed using Cox regression models adjusted for age and sex. Of the 73,294 participants without PD at the accelerometry assessment, 314 were diagnosed with PD during follow-up. Compared with those who did not develop PD, those who did had lower daily step counts (6370 vs. 4043–5585 steps/day), with greater differences observed closer to diagnosis, as well as slower walking speeds, altered step regularity, and reduced arm swing at baseline (all p < 0.001). Across groups stratified by time to PD onset, five gait measures (daily step count, maximum walking speed, step regularity, proportion of long walking bouts, and time spent walking with static arm positions) were consistently associated with subsequent PD diagnosis (all p < 0.001). Differences in real‑world gait behaviour were observable years before PD diagnosis in this large population cohort. These findings suggest that digital gait biomarkers may help characterise early motor changes detectable up to 6.8 years before a clinical diagnosis of PD.

## Introduction

Parkinson’s disease (PD) is marked by an extended prodromal phase during which striatal dopamine levels gradually decline while the condition remains below the threshold for definitive clinical diagnosis (Berg et al. 2015). This prodromal stage has a well‑recognised non‑motor phenotype including rapid eye movement (REM) sleep behaviour disorder (RBD), constipation, olfactory dysfunction, dysautonomia and anxiety (Noyce et al. 2012; Fereshtehnejad et al. 2019; Heinzel et al. 2019). The Movement Disorder Society (MDS) research criteria for prodromal PD incorporate these non‑motor symptoms, along with background risk factors, motor features, neuroimaging findings, and biomarkers, to estimate the probability of prodromal PD (Heinzel et al. 2019).

Recent research has also identified early motor abnormalities in prodromal PD, particularly subtle gait changes. Reductions in gait speed have been observed several years before clinical diagnosis (Maraki et al. 2019; Chastan et al. 2019; Din et al. 2019, 2020), and additional gait and postural abnormalities have also been reported, including slower perceptual‑motor speed, diminished trunk coordination, impaired dynamic balance, increased step-time variability, and gait asymmetry (Fereshtehnejad et al. 2019; Din et al. 2020; Amundsen-Huffmaster et al. 2021; Nisser et al. 2022; Ma et al. 2021; Postuma et al. 2006, 2012; McDade et al. 2013; Alibiglou et al. 2016). Increased step time variability and asymmetry have also been shown to predict conversion to PD in individuals with prodromal symptoms (Din et al. 2019). However, many of these findings come from cohorts at elevated risk of PD, particularly individuals with idiopathic RBD. The extent to which early gait changes occur in the general population prior to diagnosis has not been fully established.

With advances in digital medicine, wearable sensors are increasingly used in both clinical and research settings to derive digital biomarkers of motor behaviour (Brognara et al. 2019; Chan et al. 2022). These technologies allow continuous monitoring of mobility during daily life and have the potential to detect subtle motor changes associated with early disease (Din et al. 2019, 2020; Ma et al. 2021; Sanchez-Ferro et al. 2016; Weiss et al. 2015; Godinho et al. 2016). Unlike clinic‑based assessments, which provide only brief snapshots of behaviour, continuous home‑based monitoring may provide a more ecologically valid representation of real‑world gait performance (Din et al. 2020; Chan et al. 2023).

Wrist‑worn accelerometers provide a particularly scalable approach for measuring real‑world mobility at the population level (Brognara et al. 2019; Sudlow et al. 2015). Large cohort studies using UK Biobank accelerometry data have shown that global movement signals can detect PD or identify individuals who later develop PD (Schalkamp et al. 2023; Zhao et al. 2023; Williamson et al. 2021). Other work has reported associations between physical activity measures, such as step counts, and future PD diagnosis (Acquah et al. 2025). These studies demonstrate the potential of wearable accelerometry for identifying behavioural signals that precede PD diagnosis. More broadly, wearable sensor data have also been used for passive monitoring and detection of Parkinsonian motor behaviour in clinical populations (Raykov et al. 2020).

However, most previous studies have focused on either (i) global movement signatures derived from machine‑learning models or (ii) simple activity metrics such as total acceleration or step counts (Schalkamp et al. 2023; Zhao et al. 2023; Acquah et al. 2025). In contrast, much less attention has been given to detailed digital gait biomarkers that characterise the structure and quality of real-world walking behaviour. Interpretable gait measures such as walking speed, step regularity, walking bout structure, and arm movement behaviour may provide a clearer description of early motor changes associated with PD than aggregate activity measures alone. Recently, a comprehensive suite of wrist-worn sensor–derived digital gait biomarkers capturing these domains has been developed (Chan et al. 2022, 2024). This approach represents an important advance, as wrist-worn sensors are widely accepted and scalable, enabling continuous and ecologically valid assessment of gait in large populations.

In this study, we examined whether these digital gait biomarkers that characterise the structure and quality of real-world walking behaviour differ between individuals who develop PD and those who do not. The study cohort comprised more than 73,000 participants from the UK Biobank accelerometry sub-study that were followed for up to 10 years through linked electronic health records. Specifically, the aims were (i) to examine whether real‑world digital gait biomarkers capturing detailed walking behaviour are associated with future PD diagnosis, and (ii) to determine how early such changes may be detectable prior to clinical diagnosis. Understanding these behavioural signals may provide insight into the motor changes that precede PD and help inform future approaches to identifying individuals in the earliest stages of the disease.

## Methods

### Data collection

Data were obtained from the UK Biobank, a prospective cohort study comprising over 500,000 participants aged 40 to 69 years recruited between 2006 and 2010. Recruitment targeted residents living within a 40-kilometre radius of one of 22 assessment centres across the United Kingdom. Approximately 6% of invited individuals underwent baseline assessment (Fry et al. 2017).

A subsample of 103,712 individuals was invited to wear a wrist-worn device for seven days between 2013 and 2015. The accelerometer recordings were considered valid if participants wore the device for at least five days and met the UK Biobank accelerometry quality criteria for sufficient daily wear time. Of these 103,712 participants, 73,413 provided valid data for digital gait biomarker extraction and were therefore included in this analysis.

The 73,413 participants were then divided into three groups: (i) prevalent PD for those diagnosed with PD before entering the study (n = 119); (ii) incident PD for those who developed PD during the study period, i.e., between wrist accelerometry data collection and September 2023 (n = 314); and (iii) healthy controls with no PD diagnosis by the end of follow-up (n = 72,980). Definitions of prevalent and incident PD in the UK Biobank followed the hierarchical diagnostic approach, which systematically differentiate idiopathic PD from atypical parkinsonism as described by Bush et al. (Definitions of Parkinson’s Disease and the Major Causes of Parkinsonism 2018).

Individuals with incident PD were diagnosed at different time points following accelerometry data collection. To examine pre‑diagnostic changes, incident PD cases were stratified into four groups according to time from accelerometer assessment to PD diagnosis: (i) 1–40 months (*n* = 78); (ii) 41–60 months (*n* = 78); (iii) 61–80 months (*n* = 77); and (iv) 81–119 months (*n* = 81).

Participant data were linked to National Health Service (NHS) electronic health records, and entries were retrieved in January 2022. All participants provided informed consent before data collection. Ethical approval for the UK Biobank study was obtained from the NHS National Research Ethics Service.

## Digital gait biomarkers

Digital gait biomarkers were derived from wrist accelerometry data using the Watch Walk method (version 2.3), as previously reported (Chan et al. 2022, 2024). Extracting gait characteristics from wrist‑worn accelerometers is technically challenging because wrist orientation and arm movement during walking can introduce substantial non-linearity in the acceleration signal. The Watch Walk framework addresses these challenges by deriving orientation‑independent gait features and explicitly characterising arm position during walking (Chan et al. 2022, 2024).

The Watch Walk digital gait biomarker framework has previously been developed and validated in large population datasets with Walk Watch metrics shown to be associated with multiple health outcomes, including depression, cardiovascular disease, and frailty (Chan et al. 2022, 2023; Pinilla et al. 2025).

Seventeen digital gait biomarkers representing five domains of walking were extracted using signal‑processing techniques and machine‑learning regression models:

- Gait quantity: daily step counts and running duration (minutes).
- Gait speed and intensity: usual and maximum walking speed, defined as the median and 95th percentile of daily walking speed, and median cadence.
- Gait quality: step‑time variability (standard deviation of step times) and step and stride regularity calculated from the normalised autocorrelation of the filtered acceleration vector magnitude.
- Walk length distribution: longest walk duration and the cumulative percentage of walks shorter than 8 s (higher values indicate more fragmented walking) and longer than 60 s (higher values indicate longer continuous walking). The relatively short analysis windows used by the algorithm allow detection of brief walking bouts that constitute a substantial proportion of daily mobility in free-living environments. Walking bouts between 8 and 60 s represent typical short-to-moderate walking episodes during daily activity, whereas the extremes (< 8 s and > 60 s) capture fragmented and sustained walking behaviour, respectively. Previous Watch Walk analyses have shown that a higher proportion of fragmented walking (< 8 s bouts) is associated with poorer health outcomes, whereas more sustained walking (> 60 s bouts) is associated with better functional status (Chan et al. 2022, 2024; Pinilla et al. 2025).
- Hand positions during walking: percentage of walking performed with different hand positions, including hands in pockets, briefcase/grocery carrying, shoulder bag carrying (wrist above shoulder), natural arm swing and static arm positions with hands held in front of the torso and near the head. Hand positions during walking were inferred from wrist orientation and acceleration signatures during detected walking bouts using classification models developed and validated within the Watch Walk framework (Chan et al. 2022, 2024). Static arm positions, such as hands held near the head or in front of the torso, typically reflect behaviours such as phone use (e.g., talking or texting) or other activities involving limited arm movement during walking.

Hand position outcomes were also grouped into broader categories defined as the sum of the proportions of the relevant arm positions: static arm positions (hands in front of the torso and next to the head) and adaptive hand positions (hands in pocket, shoulder bag and briefcase positions). Because natural arm swing is a characteristic component of normal gait, increased time spent with static arm positions may indicate reduced arm movement during walking. In healthy individuals, static arm positions may reflect concurrent activities such as mobile phone use and object holding, whereas in PD, they may reflect motor changes, including reduced arm swing, rigidity, or bradykinesia.

### Statistical analysis

Differences in demographic characteristics between groups were assessed using chi‑square tests for sex and one‑way analyses of variance (ANOVA) with Bonferroni‑adjusted post‑hoc tests for age and body mass index.

A one-way analysis of variance (ANOVA) was used to compare differences in digital gait biomarkers among PD subgroups and controls, adjusting for age and sex. Bonferroni-adjusted post hoc tests were conducted to assess pairwise between-group differences. Variables violating the assumption of normality of residuals were transformed using standard transformations (e.g., logarithmic or square‑root transformations) where appropriate.

Effect sizes (Cohen’s D) were interpreted using the following thresholds: 0.2–0.5 small, 0.5–0.8 medium, 0.8–1.2 large and > 1.2 very large (Sawilowsky 2009).

Associations between each digital gait biomarker and time to PD diagnosis were assessed using univariable Cox regression models adjusting for age and sex. These covariates were included because they strongly influence both gait behaviour and PD risk in population studies. Additional covariates were not included to maintain model parsimony, as the primary objective was to characterise differences in behavioural gait rather than to develop a comprehensive risk prediction model.

Biomarkers that showed statistically significant associations in univariable analysis and differed between incident PD cases and controls were considered for inclusion in multivariable Cox regression models. If any two biomarkers had a correlation coefficient ≥ 0.7, only one was retained to avoid multicollinearity. This approach reduced redundancy while preserving representative variables from each gait domain.

For each digital gait domain, a separate multivariable survival model was constructed. Hazard ratios (HRs) and two‑tailed 95% confidence intervals (CIs) are reported. Standardised HRs and 95% CIs of z‑transformed biomarkers are also provided to aid interpretation. Variables violating the functional form assumption were transformed before analysis, where necessary (e.g., logarithmic or square-root transformations).

The data were analysed using IBM SPSS v.29 for Windows (SPSS Inc., Chicago, IL), and statistical significance was set at *p* < 0.05.

## Results

Table 1 presents demographics and walking measures for healthy controls and incident and prevalent PD groups, whilst Table 2 shows effect size comparisons between participants with prevalent and incident PD versus healthy controls. Both tables present parts of the same analyses: Table 1 shows the summary statistics and post hoc differences between all groups, while Table 2 presents the overall pattern and direction of effect sizes compared to healthy controls only.

**Table 1:** Demographics and walking measures for the healthy control, incident and prevalent groups. Data are mean (SD) unless specified

	Healthy control group (*n* = 73,413)	Incident PD (time to diagnosis)				Prevalent PD (*n* = 119)	*p*-value for ANOVAs
		1 to 40 months (*n* = 78)	41 to 60 months (*n* = 78)	61 to 80 months (*n* = 77)	81 to 119 months (*n* = 81)		
*Demographics*							
Age, years	61.8 (7.8)	68.3 (5.6)	68.6 (5.4)	68.4 (4.9)	68.4 (4.9)	67.0 (6.3)	**< 0.001** ^a, c,e, g,i^
Body Mass Index, kg/m2	26.74 (4.53)	26.94 (4.25)	27.16 (4.80)	26.97 (4.37)	27.20 (4.23)	26.29 (4.79)	0.691
Sex, male (%)	33,496 (45.9)	59 (75.6)	55 (70.5)	57 (74.0)	52 (64.2)	70 (58.8)	**< 0.001**
*Gait quantity*							
Average step count, steps/day	6370 (2656)	4043 (2017)	4719 (2555)	5397 (2918)	5585 (2173)	4212 (2622)	**< 0.001** ^b, d,j, k,l, p,q^
Average run duration, minutes	4.47 (6.38)	2.16 (4.06)	2.10 (3.22)	3.11 (5.15)	3.47 (7.27)	2.40 (3.29)	**0.001**
Gait speed and intensity							
Average maximum speed, cm/s	149.3 (8.2)	144.4 (10.4)	145.1 (9.6)	148.9 (9.6)	146.3 (9.2)	144.4 (10.0)	**< 0.001** ^b, d,h, j,k, o,p^
Average usual speed, cm/s	114.5 (7.9)	110.3 (10.4)	110.8 (8.2)	114.7 (9.6)	112.3 (8.3)	109.5 (7.9)	**< 0.001** ^b, d,j, k,o, p,q^
Average cadence, median	100.8 (3.2)	100.21 (3.6)	100.9 (3.1)	101.3 (2.7)	101.0 (3.6)	102.1 (5.3)	**< 0.001** ^i, n^
*Gait quality*							
Average step time variability best	60 (14)	65 (19)	61 (15)	61 (15)	60 (16)	61 (15)	0.135
Step regularity	65.4 (4.7)	65.6 (6.2)	66.5 (4.3)	66.8 (5.7)	65.9 (6.2)	63.1 (6.7)	**< 0.001** ^c, e,j, m,p, q,s^
Stride regularity	52.4 (4.6)	52.4 (5.7)	52.9 (4.2)	53.1 (5.0)	53.4 (5.6)	49.5 (6.8)	**< 0.001** ^j, m,p, q,s^
*Walk length distribution*							
Average longest walk, seconds	218.5 (139.5)	196.6 (133.5)	187.9 (115.0)	242.4 (220.7)	210.5 (118.8)	138.0 (117.9)	**< 0.001** ^j, m,p, q^
Walks **≤** 8s, %	39 (56)	42 (82)	41 (60)	40 (71)	39 (58)	38 (87)	**< 0.001** ^a, c,m, s^
Walks **≥** 60s, %	2.1 (1.7)	3.2 (3.3)	2.6 (2.2)	3.0 (2.5)	2.3 (1.9)	1.5 (1.8)	**< 0.001** ^a, e,j, m,p, q,s^
*Walk hand positions (%)*							
Shoulder bag	4.64 (4.56)	7.87 (9.93)	5.07 (5.24)	7.31 (8.27)	5.63 (7.12)	4.85 (6.60)	**< 0.001** ^a, e,m, p,r, w,ab^
Hands in pocket	1.36 (1.57)	0.89 (1.16)	1.16 (1.46)	1.22 (1.54)	1.00 (1.50)	0.91 (1.03)	0.267
Briefcase	1.89 (2.15)	2.13 (2.47)	2.02 (2.38)	2.35 (2.25)	2.46 (2.97)	2.05 (2.73)	**< 0.001** ^e, g^
Natural arm swing	90.7 (7.1)	86.8 (12.1)	89.7 (7.7)	87.7 (10.3)	88.9 (10.3)	89.7 (9.3)	**< 0.001** ^b, d,f, h,n^
Hands near head	0.71 (1.18)	0.78 (1.00)	0.89 (1.48)	0.62 (0.94)	1.28 (2.33)	1.16 (1.93)	**< 0.001** ^g, i,u, aa,^
Hands front of torso	0.59 (0.61)	1.46 (2.36)	1.13 (1.19)	0.73 (0.58)	0.64 (0.50)	1.24 (1.39)	**< 0.001** ^a, c,i, m,r, u,v, w,x, y,z^

Post-hoc Bonferroni comparisons with age and sex as co-variates, p < 0.05: ^a^time to diagnosis, 0 to 40 months > healthy control; ^b^healthy control > time to diagnosis, 0 to 40 months; ^c^time to diagnosis, 41 to 60 months > healthy control; ^d^healthy control > time to diagnosis, 41 to 60 months; ^e^time to diagnosis, 61 to 80 months > healthy control; ^f^healthy control > time to diagnosis, 61 to 80 months; ^g^time to diagnosis, 81 to 119 months > healthy control; ^h^healthy control > time to diagnosis, 81 to 119 months; ^i^Parkinson’s disease > healthy control; ^j^healthy control > Parkinson’s disease; ^k^time to diagnosis, 61 to 80 months > time to diagnosis, 0 to 40 months; ^l^time to diagnosis, 81 to 119 months > time to diagnosis, 0 to 40 months; ^m^Parkinson’s disease < time to diagnosis, 0 to 40 months; ^n^Parkinson’s disease > time to diagnosis, 0 to 40 months; ^o^time to diagnosis, 61 to 80 months > time to diagnosis, 41 to 60 months; ^p^Parkinson’s disease < time to diagnosis, 61 to 80 months; ^q^Parkinson’s disease < time to diagnosis, 81 to 119 months; ^r^time to diagnosis, 0 to 40 months > time to diagnosis, 81 to 119 months; ^s^Parkinson’s disease < time to diagnosis, 41 to 60 months; ^u^Parkinson’s disease > time to diagnosis, 61 to 80 months; ^v^Parkinson’s disease > time to diagnosis, 81 to 119 months; ^w^time to diagnosis, 0 to 40 months > time to diagnosis, 41 to 60 months; ^x^time to diagnosis, 0 to 40 months > time to diagnosis, 61 to 80 months; ^y^time to diagnosis, 41 to 60 months > time to diagnosis, 81 to 119 months; ^z^time to diagnosis, 41 to 60 months > Parkinson’s disease; ^aa^time to diagnosis, 81 to 119 months > time to diagnosis, 61 to 80 months; ^ab^time to diagnosis, 41 to 60 months < time to diagnosis, 61 to 80 months. Significant p-values bolded.

## Comparisons between prevalent PD and healthy controls

In comparison to healthy controls, the prevalent PD group took significantly (*p* ≤ 0.05) fewer average steps per day, slower average usual walking speed, slower maximum walking speed, increased average cadence, lower step counts and worse stride regularity. The prevalent PD group also had shorter average durations of their longest walks, a lower proportion of walks over 60 s, and a higher proportion of walks with static arm positions with hands held near the head and in front of the torso.

**Table 2:**
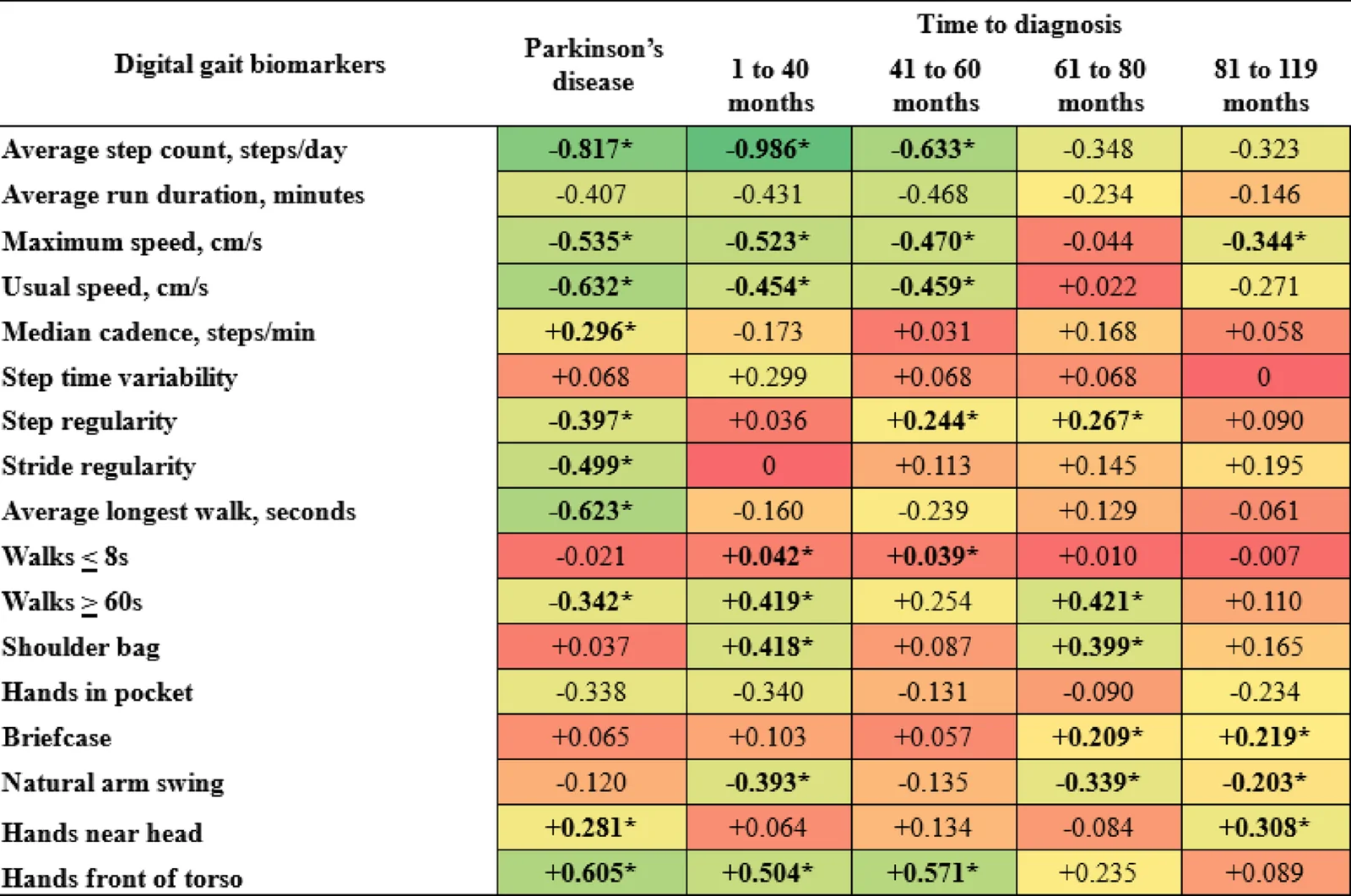
Heatmap visualising the overall pattern of effect sizes and direction in people with prevalent and incidental PD, versus healthy controls

Significant effect sizes (bolded *, p<0.001) from the Table 1 data and presenting effect sizes (color coded from red 0.0 to green ±1.0) versus healthy controls only. Direction (+) indicates Prevalent/Incident PD > than Healthy Controls, and (-) indicates Prevalent/Incident PD < than Healthy Controls.

### Comparisons between incident PD subgroups and healthy controls

Compared to the healthy controls, individuals in each incident PD subgroup exhibited distinct gait characteristics across multiple domains. In terms of gait quantity, those diagnosed within 1 to 40 months, and 41 to 60 months took significantly fewer average steps per day, with large (Cohen’s d = 0.986) and moderate (Cohen’s d = 0.633) effect sizes, respectively. Regarding gait speed and intensity, slower maximum walking speeds were observed in individuals diagnosed between 1 and 40 months (Cohen’s d = 0.523), 41 and 60 months (Cohen’s d = 0.470), and 81 and 119 months (Cohen’s d = 0.344). Additionally, slower average walking speeds were found in the 1- to 40-month (Cohen’s d = 0.454) and 41- to 60-month (Cohen’s d = 0.459) groups.

Gait quality differences were also evident, with higher step regularity in individuals diagnosed between 41 and 60 months (Cohen’s d = 0.244) and 61 and 80 months (Cohen’s d = 0.267), suggesting more rhythmic but potentially less adaptive walking patterns. For walk length distribution, a higher proportion of short walks (less than 8 s) was observed in the 1 to 40 month (Cohen’s d = 0.042) and 41 to 60 month (Cohen’s d = 0.039) groups, while a greater proportion of long walks (over 60 s) was evident in the 1 to 40 month (Cohen’s d = 0.419) and 61 to 80 month (Cohen’s d = 0.421) groups.

Differences in hand position while walking were also notable. Individuals diagnosed within 1 to 40 months showed a higher proportion of walking with a shoulder bag (Cohen’s d = 0.418) and hands held in front of the torso (Cohen’s d = 0.504), and a lower proportion of walking with natural arm swing (Cohen’s d = 0.393), indicating a greater prevalence of static arm positions during walking. Those diagnosed from 41 to 60 months exhibited more walking with hands held statically in front of the torso (Cohen’s d = 0.571). The 61- to 80-month group showed increased walking while carrying a shoulder bag (Cohen’s d = 0.399) and a briefcase (Cohen’s d = 0.209), along with reduced arm swing (Cohen’s d = 0.339). Finally, individuals diagnosed from 81 to 119 months had a higher proportion of walking while carrying a briefcase (Cohen’s d = 0.219) and with hands held near the head (Cohen’s d = 0.308), and a lower proportion of walking with arm swing (Cohen’s d = 0.203).

### Comparisons between incident PD subgroups and prevalent PD

Compared to individuals with prevalent PD, each incident PD subgroup demonstrated distinct walking characteristics across multiple gait domains. In terms of gait quantity, individuals diagnosed at 61 to 80 months and 81 to 119 months had a higher average number of steps per day. For gait speed and intensity, those diagnosed from 61 to 80 months showed higher maximum walking speeds, while both the 61 to 80 month and 81 to 119 month groups demonstrated greater average usual walking speeds. In contrast, individuals diagnosed within 1 to 40 months presented with a lower average cadence.

Gait quality differences were also observed, with higher step and stride regularity in individuals diagnosed within 1 to 40 months, 61 to 80 months, and 81 to 119 months. Regarding walk length distribution, individuals diagnosed from 1 to 40 months, 61 to 80 months, and 81 to 119 months exhibited longer average longest walks and a higher proportion of walks exceeding 60 s. A higher proportion of short walks (less than 8 s) was observed only in the 1–40-month group.

Differences in hand position during walking were also evident. Individuals diagnosed within 1 to 40 months showed a higher proportion of walking with a shoulder bag and hands held statically in front of the torso, and a lower proportion of walking with arm swing. Those diagnosed from 41 to 60 months exhibited a higher proportion of walking with hands held in front of the torso. The 61- to 80-month group showed increased walking while carrying a shoulder bag and reduced walking with hands held next to the head or in front of the torso. Finally, individuals diagnosed between 81 and 119 months had a lower proportion of walking with hands held statically in front.

### Cox regression of digital gait biomarkers predicting incident PD

Table 3 presents the results of Cox regression analyses examining the predictive value of digital gait biomarkers for incident PD. In the univariate models, adjusted for age and sex, several gait features were significantly associated with PD incidence across multiple domains. These included gait quantity, gait speed and intensity, gait quality, walk length distribution (specifically, the proportion of walking bouts exceeding 60 s), and hand positions during walking, particularly static arm positions with hands held next to the head or in front of the torso. Shoulder bag and briefcase positions did not show predictive value. In the multivariate analysis (Table 4), which included age and sex as covariates, fewer steps per day, slower maximum walking speed, lower step regularity, a higher proportion of walks exceeding 60 s, and walking with the hand in a static position remained significant predictors of incident PD.

**Table 3:** Cox regression analyses examining the predictive value of digital gait biomarkers for incident PD

	Hazard ratio	Confidence interval (95%)	Hazard ratio for standardised variables	Confidence interval (95%)	*p*-value
*Gait quantity*					
Average step count, steps/day	0.98	(0.98 to 0.98)	0.95	(0.94 to 0.96)	**< 0.001**
Average run duration, minutes^a^	0.97	(0.96 to 0.97)	0.97	(0.96 to 0.97)	**< 0.001**
*Gait speed and intensity*					
Average maximum speed, cm/s	0.99	(0.99 to 1.00)	0.95	(0.94 to 0.96)	**< 0.001**
Average usual speed, cm/s	1.00	(1.00 to 1.00)	0.99	(0.98 to 0.99)	**< 0.001**
Average cadence, median	1.00	(1.00 to 1.00)	1.00	(0.99 to 1.01)	0.980
*Gait quality*					
Average step time variability best	1.00	(1.00 to 1.00)	1.03	(1.02 to 1.03)	**< 0.001**
Step regularity	0.99	(0.99 to 0.99)	0.97	(0.96 to 0.98)	**< 0.001**
Stride regularity	0.99	(0.99 to 1.00)	0.97	(0.97 to 0.98)	**< 0.001**
*Walk length distribution*					
longest walk duration, seconds^a^	0.98	(0.97 to 0.99)	0.99	(0.98 to 0.99)	**< 0.001**
Walks **≤** 8s	1.00	(1.00 to 1.00)	1.00	(0.99 to 1.01)	0.910
Walks **≥** 60s^b^	1.25	(1.10 to 1.42)	1.01	(1.01 to 1.02)	**< 0.001**
*Walk hand positions*					
Shoulder bag	1.00	(1.00 to 1.00)	1.00	(0.99 to 1.01)	0.900
Hands in pocket	1.01	(1.00 to 1.01)	1.01	(1.01 to 1.02)	**< 0.001**
Briefcase	1.00	(1.00 to 1.00)	1.00	(1.00 to 1.01)	0.510
Natural arm swing	1.00	(1.00 to 1.00)	0.98	(0.97 to 0.98)	**< 0.001**
Hands near head	1.07	(1.07 to 1.08)	1.09	(1.08 to 1.10)	**< 0.001**
Hands front of torso	1.08	(1.07 to 1.09)	1.05	(1.04 to 1.06)	**< 0.001**
Static arm positions (hands near head + front of torso)	1.06	(1.06 to 1.07)	1.10	(1.09 to 1.10)	**< 0.001**
Adaptive hand positions (shoulder + pocket + briefcase)	1.00	(1.00 to 1.00)	1.00	(1.00 to 1.01)	0.240

Includes age and sex as covariates

^a^The natural logarithm of the digital gait biomarker was used in the Cox regression model to satisfy the functional form assumption. ^b^The square root of the digital gait biomarker was used in the Cox regression model to satisfy the functional form assumption. Significant p-values bolded.

**Table 4:** Multivariate Cox regression analyses examining the predictive value of digital gait biomarkers from multiple domains for incident PD

	Hazard ratio	Confidence interval (95%)	Hazard ratio for standardized variables	Confidence interval (95%)	*p*-value
*Gait quantity*					
Average step count, steps/day	0.99	(0.98 to 0.99)	0.96	(0.95 to 0.97)	**< 0.001**
*Gait speed and intensity*					
Average maximum speed, cm/s	1.00	(1.00 to 1.00)	0.97	(0.96 to 0.98)	**< 0.001**
*Gait quality*					
Step regularity	0.99	(0.99 to 0.99)	0.95	(0.95 to 0.96)	**< 0.001**
*Walk length distribution*					
Walks **≥** 60s	1.03	(1.03 to 1.04)	1.06	(1.05 to 1.07)	**< 0.001**
*Walk hand positions*					
Static Arm Positions (hands near head + front of torso)	1.06	(1.06 to 1.07)	1.10	(1.09 to 1.10)	**< 0.001**

One variable for each digital gait biomarker included in the model; age and sex included as covariates

^a^The square root of the digital gait biomarker was used in the Cox regression model to satisfy the functional form assumption. Significant p-values bolded.

## Discussion

### Population-scale gait changes preceding PD diagnosis

In this study of 73,413 community-dwelling older adults, we observed population-level differences in real-world gait behaviour among individuals who were later diagnosed with PD. These differences were detectable several years prior to diagnosis and were characterised by gait changes, and some of these gait characteristics, particularly arm movement patterns (e.g., reduced natural arm swing and hands in a static position), diverged from those of healthy controls up to 6.8 years before clinical diagnosis. Across the different pre-diagnostic time windows examined, five digital gait measures consistently differed between individuals who later developed PD and those who did not: daily step count, maximum walking speed, step regularity, the proportion of long walking bouts, and the proportion of time spent walking with static arm positions. Together, these findings suggest that subtle changes in real-world walking behaviour may precede clinical diagnosis of PD in large population cohorts, providing new insights into early motor changes associated with the disease.

### Interpretation of gait characteristics

We observed a stepwise decline in daily step counts beginning up to 6.8 years prior to PD diagnosis. Compared with individuals who did not develop PD, those who later developed PD exhibited lower daily step counts (6,370 vs. 4043–5585 steps/day), with step counts decreasing progressively as the time to diagnosis shortened. The differences in daily step counts were 785 to 2327 steps/day lower than in healthy controls across the pre-diagnostic groups. All of these differences exceeded the recently proposed minimal clinically important difference (MCID) of 581 steps/day for average daily steps in people with mild-to-moderate PD (Bianchini et al. 2025). The differences observed in prevalent PD and in individuals within 60 months of diagnosis also exceeded the higher thresholds of 1200 steps/day based on clinical and health-related measures and 1592 steps/day based on patient-reported outcomes (Bianchini et al. 2025).

Several factors could contribute to the observed reduction in daily step counts. Subjective gait impairment and postural difficulties, often reported before formal diagnosis, likely diminish individuals’ capacity and motivation to engage in prolonged walking (Friederich et al. 2019). Additionally, neuropsychiatric symptoms, including apathy, depression and anxiety, have been reported in prodromal PD and may play a significant role in reducing physical activity (Jacob et al. 2010; Waele et al. 2022). The daily step count was lowest in individuals 0 to 40 months before diagnosis, even lower than in those with an established PD diagnosis, and the usual gait speed in individuals 0 to 60 months before diagnosis was relatively similar to that of people diagnosed with PD. This pattern may reflect the initiation of dopaminergic therapy in individuals diagnosed with the condition, which is expected to improve motor symptoms and, consequently, increase physical activity.

Individuals who later developed PD *also* demonstrated reduced motor reserve, as reflected in slower maximum gait speed than healthy controls, up to 5 years before diagnosis. This finding is consistent with evidence that people with PD have difficulty adjusting their gait under time constraints (Pelicioni et al. 2019). Because speed reflects displacement over time, disruptions in temporal gait regulation may indicate early motor changes associated with PD (Bernardinis et al. 2019). While slower gait speed, assessed in clinical settings, has been consistently reported as an early motor sign of prodromal PD (Maraki et al. 2019; Chastan et al. 2019), most studies have focused on individuals at high risk for PD, such as mutation carriers or those with idiopathic REM sleep behaviour disorder (Din et al. 2020; Postuma et al. 2012; Mirelman et al. 2016).

For average walking speed measured in daily life, the differences from healthy controls were comparatively modest, ranging from 2.2 to 5.0 cm/s lower in most PD groups. These differences were below the published MCID of 8.2 cm/s for gait velocity in people with PD (Baudendistel et al. 2024). However, that threshold was derived from medicated individuals with established PD walking on an instrumented walkway and may not be directly transferable to average or maximum walking speed measured continuously under free-living conditions. Similarly, MCIDs are currently unavailable for the other digital gait measures evaluated in this study, highlighting the need for future research to establish consensus on clinically meaningful thresholds for many free-living gait metrics.

Individuals who later developed PD also spent more time walking with a static arm position, with hands in front of the torso, such as holding a shoulder bag, and less time walking with a natural arm swing, indicating reduced arm movement during gait. These findings extend those of Mirelman et al., who proposed arm swing asymmetry as an early biomarker of prodromal PD in LRRK2 mutation carriers (Mirelman et al. 2016). While static arm postures during walking can occur in healthy individuals when the hands are engaged in concurrent activities such as holding objects or using a mobile phone, in PD, they may instead reflect early motor features, including reduced arm swing, rigidity, or bradykinesia. The long lead time of these changes suggests that subtle alterations in arm movement control may emerge well before overt clinical symptoms and therefore represent an informative behavioural marker of early motor changes associated with PD.

The finding of higher step regularity in some pre-diagnostic groups warrants cautious interpretation. One possible interpretation is that increased step regularity reflects reduced variability in motor output, which may indicate diminished adaptability of gait control. Such changes may represent early alterations in the neural control of locomotion before overt gait impairment becomes clinically evident.

### Comparison with previous accelerometry studies

Traditional pedometry and most previous analyses of data from wrist-worn devices have relied on aggregate activity metrics or machine-learning features, which may be relatively device-orientation-agnostic (Schalkamp et al. 2023; Zhao et al. 2023; Williamson et al. 2021; Acquah et al. 2025). A methodological challenge when analysing gait from wrist‑worn accelerometers is that wrist orientation and arm movement can introduce substantial non-linearity and variability in the recorded acceleration signal. The Watch Walk framework was developed to address these issues by explicitly modelling arm position during walking (Chan et al. 2022, 2024). In addition, the relatively short analysis windows used by the Watch Walk algorithm allow detection of brief walking bouts that constitute a large proportion of daily mobility in free‑living environments. These enhanced methodological features enable a more detailed behavioural characterisation of real‑world gait behaviour from wrist‑worn sensors.

In a previous longitudinal study, Del Din et al. showed prodromal gait changes may occur up to four years before diagnosis in individuals who later converted to PD (Din et al. 2019). Building on that work, the present study, in a larger population of 73,413 individuals, demonstrated that alterations in arm movement during walking may occur up to 6.8 years prior to clinical diagnosis.

### Broader implications

Our results suggest that the motor prodrome in idiopathic PD may involve reductions in arm swing, gait speed, and daily step count. Importantly, this motor phenotype is detectable in a large, real-world population. Irrespective of the timeframe, five gait-related outcomes consistently distinguished individuals who later developed PD from healthy controls: fewer steps per day, slower maximum walking speed, poorer step regularity, more frequent walking bouts within 60 seconds, and a tendency to hold the hand in a static position while walking.

With more than 50 ongoing trials investigating potential disease-modifying therapies targeting pathways including alpha-synuclein, glucocerebrosidase, GLP-1, neuroinflammation, mitochondrial function and others (McFarthing et al. 2024), and clinical and research interest in precision medicine, including on the use of wearables, a better understanding of the behavioural changes that precede clinical diagnosis may therefore be valuable for future risk-enrichment strategies. Future research should examine the value of repeated assessments of these markers to improve stratification of individuals at risk of developing more severe motor phenotypes of PD and to optimise the timing and targeting of therapeutic interventions.

### Strengths and limitations

This study has several strengths. Its large sample size and continuous monitoring of walking behaviour under free-living conditions enabled the investigation of gait characteristics across multiple pre-diagnostic time windows, providing insights into early motor differences that are difficult to capture in smaller clinical cohorts. The digital gait biomarkers were derived using a framework previously validated in large populations (Chan et al. 2022), supporting their use as objective measures of real-world walking behaviour. In addition, linkage to an extensive database containing detailed sociodemographic and health information allowed participants to be followed for up to 10 years after accelerometry assessment.

Several limitations should also be considered. First, although a number of gait measures differed consistently between groups, the magnitude of some associations was modest. This is not unexpected in a population-based cohort, in which pre-diagnostic motor differences are likely to be subtle and heterogeneous.

Second, the timing and classification of PD diagnoses were based on linked electronic health records and ICD-10 codes, which may be incomplete or inaccurate. Clinical diagnoses could not be independently confirmed, and the recorded date of diagnosis may not correspond precisely to the onset of clinically recognisable disease. Several gait characteristics examined in this study may also occur in atypical parkinsonian disorders. Although participants with recorded atypical diagnoses were excluded, incomplete coding may have resulted in some participants being misclassified, while others with undiagnosed PD may have been included in the comparison group.

Third, detailed clinical information on factors that may influence free-living gait was not consistently available or incorporated into the analyses. These factors may include transient, pain and medication use, other conditions, and the use of walking aids. These factors may have contributed to variability in the observed measures. Fourth, accelerometry was obtained at a single assessment, and the study therefore compared groups at different intervals before diagnosis rather than measuring within-person gait change over time.

Finally, participants were required to provide at least five valid days of wrist-sensor data. This requirement, together with the recognised healthy-volunteer characteristics of the UK Biobank cohort, may have selected a healthier, more mobile, and more motivated group than the wider population of older adults. Further external validation is required before these digital gait biomarkers can be considered standalone predictors of PD or applied to individual clinical decision-making.

## Conclusions

This study demonstrates that differences in real‑world gait behaviour, including reduced arm movement, slower walking speed and lower walking activity, may be observed years before PD diagnosis in a large population cohort. By analysing digital gait biomarkers derived from wrist‑worn accelerometry, this study highlights the potential of real‑world mobility monitoring to improve understanding of early motor alterations in PD. As wrist-worn devices are widely accepted and scalable, these digital gait biomarkers may represent an important advance in continuous, ecologically valid, large-scale assessment of real-world gait. Future studies should include prodromal cohorts in within-individual longitudinal trajectories to confirm our findings and investigate how these metrics develop over time.

## Data Availability

The data used in this study were obtained from the UK Biobank under application number 103840. These data are available to bona fide researchers upon application to the UK Biobank (https://www.ukbiobank.ac.uk/), subject to approval and data access policies. Accelerometry data were processed using the Watch Walk algorithm (v2.3.6), which is freely available to research groups via https://watchwalk.neura.edu.au/.
